# Exploring the influence of circulating endocannabinoids and nucleus accumbens functional connectivity on anorexia nervosa severity

**DOI:** 10.1038/s41380-023-02253-2

**Published:** 2023-09-28

**Authors:** Romina Miranda-Olivos, Isabel Baenas, Trevor Steward, Roser Granero, Antoni Pastor, Isabel Sánchez, Asier Juaneda-Seguí, Amparo del Pino-Gutiérrez, José A. Fernández-Formoso, Nuria Vilarrasa, Fernando Guerrero-Pérez, Nuria Virgili, Rafael López-Urdiales, Susana Jiménez-Murcia, Rafael de la Torre, Carles Soriano-Mas, Fernando Fernández-Aranda

**Affiliations:** 1 Clinical Psychology Unit, Bellvitge University Hospital, L’Hospitalet de Llobregat, 08907 Barcelona, Spain; 2 Ciber Fisiopatología de la Obesidad y Nutrición (CIBERObn), Instituto de Salud Carlos III, 08907 Barcelona, Spain; 3 Psychoneurobiology of Eating and Addictive Behaviors Research Group, Neurosciences Program, Bellvitge Biomedical Research Institute (IDIBELL), 08908 Barcelona, Spain; 4 Doctoral Program in Medicine and Translational Research, University of Barcelona, 08036 Barcelona, Spain; 5 Melbourne School of Psychological Sciences, Faculty of Medicine, Dentistry and Health Sciences, University of Melbourne, Parkville, VIC 3010 Australia; 6 Department of Psychobiology and Methodology, Autonomous University of Barcelona, 08193 Barcelona, Spain; 7 Integrative Pharmacology and Systems Neuroscience research group, Hospital del Mar Research Institute (IMIM), 08003 Barcelona, Spain; 8 Ciber de Salud Mental (CIBERSAM), Instituto Salud Carlos III, 28029 Barcelona, Spain; 9 Department of Psychiatry, Bellvitge University Hospital-IDIBELL, C/Feixa Llarga s/n, 08907 Barcelona, Spain; 10 Department of Public Health, Mental Health and Perinatal Nursing, School of Nursing, University of Barcelona, 08907 Barcelona, Spain; 11 Department of Endocrinology and Nutrition, Bellvitge University Hospital-IDIBELL, C/Feixa Llarga s/n, 08907 Barcelona, Spain; 12 CIBERDEM-CIBER de Diabetes y Enfermedades Metabólicas Asociadas, Instituto de Salud Carlos III, C/Monforte de Lemos 3-5, 28029 Madrid, Spain; 13 Department of Clinical Sciences, School of Medicine and Health Sciences, University of Barcelona, 08907 Barcelona, Spain; 14 Department of Experimental and Health Sciences, Pompeu Fabra University (CEXS-UPF), 08002 Barcelona, Spain; 15 Department of Social Psychology and Quantitative Psychology, School of Psychology, University of Barcelona, 08035 Barcelona, Spain

**Keywords:** Neuroscience, Psychiatric disorders

## Abstract

Anorexia nervosa (AN) is a severe psychiatric disorder characterized by a harmful persistence of self-imposed starvation resulting in significant weight loss. Research suggests that alterations in the nucleus accumbens (NAcc) and circulating endocannabinoids (eCBs), such as anandamide (AEA) and 2-arachidonoylglycerol (2-AG), may contribute to increased severity and maladaptive behaviors in AN, warranting an examination of the interplay between central reward circuitry and eCBs. For this purpose, we assessed NAcc functional connectivity and circulating AEA and 2-AG concentrations in 18 individuals with AN and 18 healthy controls (HC) to test associations between circulating eCBs, NAcc functional connectivity, and AN severity, as defined by body mass index (BMI). Decreased connectivity was observed between the NAcc and the right insula (NAcc-insula; *p*_FWE_ < 0.001) and the left supplementary motor area (NAcc-SMA; *p*_FWE_ < 0.001) in the AN group compared to HC. Reduced NAcc-insula functional connectivity mediated the association between AEA concentrations and BMI in the AN group. However, in HC, NAcc-SMA functional connectivity had a mediating role between AEA concentrations and BMI. Although no significant differences in eCBs concentrations were observed between the groups, our findings provide insights into how the interaction between eCBs and NAcc functional connectivity influences AN severity. Altered NAcc-insula and NAcc-SMA connectivity in AN may impair the integration of interoceptive, somatosensory, and motor planning information related to reward stimuli. Furthermore, the distinct associations between eCBs concentrations and NAcc functional connectivity in AN and HC could have clinical implications for weight maintenance, with eCBs being a potential target for AN treatment.

## Introduction

Anorexia nervosa (AN) is an eating disorder (ED) characterized by food restriction, body image disturbances, and a harmful drive for weight loss despite negative consequences [1–4]. Significant weight loss has been shown to have increasingly pronounced adverse effects on both mental and physical well-being as individuals with AN continue to lose weight [5–7]. As such, the Diagnostic and Statistical Manual of Mental Disorders, fifth edition (DSM-5) categorizes the severity of AN based on body mass index (BMI), ranging from mild (BMI ≥ 17 kg/m²) to extreme (BMI < 15 kg/m²) [8].

Aberrant eating behavior in AN is believed to be underpinned by dysfunctional reward processing and diminished response to homeostatic signals [9–11]. Neuroimaging and behavioral research have identified alterations in brain reward and cognitive control systems in patients with AN [9, 11–15], with initial evidence suggesting brain activation and connectivity measures may be associated with AN treatment outcomes [16, 17].

The nucleus accumbens (NAcc) is a crucial region for assessing rewards. It receives dopaminergic projections from various brain areas, including the ventral tegmental area (VTA), prefrontal cortex, insula, amygdala, and lateral hypothalamus [18–20]. These projections play a role in metabolic homeostasis and influence both the motivation to eat (“wanting”) and the hedonic evaluation of food preferences (“liking”) [21–25]. Both psychological processes (i.e., “wanting and liking”) have been described by Berridge [26] as mesolimbic processes associated with the representation of rewarding stimuli. The valuation of an external stimulus perceived as rewarding increases motivation and approach to it, just as cognitive processing can increase the desire for an external rewarding stimulus in its absence.

Studies in individuals with AN have found decreased activity in the NAcc and insula in response to taste stimuli compared to healthy controls (HC) [27]. Although patients with AN respond to hedonic stimuli (such as palatable food), they report not “wanting” to be motivated by these types of stimuli for fear of gaining weight [9, 27, 28]. By contrast, when viewing underweight body images, patients with AN exhibited increased NAcc activation [29, 30]. NAcc alterations have been associated with the AN severity [31] and can persist even after weight recovery [32], suggesting that this region contributes to aberrant reinforcement, promoting behaviors such as starvation or over-exercising. For this reason, the NAcc has been used as a therapeutic target for deep brain stimulation (DBS) in AN [17, 33]. Preliminary results have demonstrated NAcc DBS can help improve AN treatment outcomes [17] and to reduce depressive and anxious symptoms [33].

A growing body of evidence supports the implication of the endocannabinoid system (eCB system) in the brain reward circuitry. The eCB system is composed of endogenous cannabinoid (CB) receptors type 1 and 2 (CB1R and CB2R) expressed in the central and the peripheral nervous system and two main lipidic endogenous ligands: anandamide (AEA) and 2-arachidonoylglycerol (2-AG) [34]. In the brain, the endocannabinoids (eCBs) are released from VTA to the NAcc, the amygdala, and the frontal cortex [35, 36], modulating extracellular levels of dopamine in the NAcc [24, 37]. Most studies focused on the eCB system have converged on describing its involvement in wanting and liking processes [25, 26]. For instance, studies in animals have demonstrated that an injection of 2-AG in the NAcc promotes orexigenic function in the lateral hypothalamus via inhibition of GABAergic signaling in rats [38, 39].

Based on this knowledge, some pharmacological studies have focused on the CB1R as a potential target in the treatment of AN, reporting limited evidence regarding safety and efficacy [40–42]. For instance, some interventional studies have tested the efficacy of a synthetic cannabinoid called dronabinol in patients with AN [40, 42]. This compound acts as a CB1R agonist, promoting appetite. While initial results did not show a significant increase in body weight and revealed adverse effects in some patients [42], a subsequent study demonstrated a modest weight gain without tolerability problems [40]. It is currently considered a potential pharmacological option for AN treatment albeit with limited evidence regarding its effectiveness [41].

Research focused on exploring the eCB system in AN has provided mixed results. While some studies comparing individuals with AN and HC reported elevated AEA concentrations in AN [14, 43], other research has identified decreased AEA concentrations [13] or no significant differences [44]. These alterations in AEA concentrations observed in AN were maintained even after weight restoration [13, 14]. Regarding 2-AG, most studies have found no significant differences comparing patients with AN and HC [13, 14, 43, 44]. Likewise, studies investigating the activity and expression of CB receptors have also shown inconsistent results. For instance, a study observed CB1R up-regulation in patients with AN and BN compared to HC [45]. Frieling et al. [45] reported a negative association between CB1R availability and ED severity, with higher symptomatology being associated with lower CB1R expression [45]. However, another study reported down-regulation only in patients with EDs presenting self-injurious behavior compared to those who did not report such behavior and HC [46]. Despite these divergent results, both studies converged in linking a down-regulation of CB1R with a more severe presentation of the disorder.

Neuroimaging studies support that the eCB system can mediate brain reward circuits involved in the control of appetite and motivation toward food. A recent study in HC reported that circulating AEA concentrations were associated with functional connectivity between the lateral hypothalamus and the ventral striatum during satiety and between the anterior cingulate cortex and the insula during fasting [47]. In EDs, one positron emission tomography study (PET) investigated CB1R availability in the brain of patients with AN and observed an increased availability in the insula in AN and BN compared with HC [48], as well as in the frontal and temporal lobes. Gérard et al. [48] hypothesized that this up-regulation of the CB1R in the insular, frontal, and temporal regions in AN could represent a compensatory mechanism in response to altered concentrations of circulating eCBs as a consequence of chronic hunger.

A deeper understanding of the interaction between the brain reward system and the eCB system could help to better elucidate the modulation that they exert on motivational and homeostatic processes related to altered food intake in AN. Likewise, the identification of the neurobiological substrates of maladaptive behavior in AN could allow for the delineation of potential therapeutic targets aimed at counteracting their impact. As such, the present study sought to investigate the intrinsic functional architecture of the NAcc using resting-state functional magnetic resonance imaging (fMRI) and fasting circulating AEA and 2-AG concentrations in individuals with AN compared to HC. We aimed to explore whether circulating eCBs concentrations would have an influence on NAcc functional connectivity and BMI, as a measure of AN severity [8]. We hypothesized that individuals with AN would exhibit reduced NAcc resting-state functional connectivity (rsFC) with prefrontal regions, the insula, temporal, and parietal regions, compared with HC. Likewise, we expected alterations in the functional connectivity patterns of the NAcc and eCBs concentrations would influence AN severity.

## Methods

### Participants

A cross-sectional study was conducted on 36 adult women (18–47 years old): 18 individuals belonging to AN (BMI < 18 kg/m^2^) and 18 HC (BMI = 18–24.99 kg/m^2^). All patients in the AN group were diagnosed with restrictive subtype (AN-R) using DSM-5 criteria [8] based on a semi-structured interview (SCID-5) [49] carried out by experienced clinical psychologists and psychiatrists. The selection of a clinical sample composed exclusively of patients with AN-R aimed to focus on a specific subtype of AN and to minimize the potential effect of purging behaviors on eCBs concentrations [43]. Likewise, BMI was used as a severity criterion in AN according to the DSM-5 [8]. Patients were recruited between 2016 and 2021 from the Eating Disorders Unit at the Bellvitge University Hospital (Barcelona, Spain). All patients were admitted to a three-month day-hospital treatment program consisting of refeeding treatment and daily group cognitive-behavioral therapy (CBT) sessions. The HC group was recruited via advertisements from the same catchment area.

In order to detect the presence of a psychiatric disorder, all participants were evaluated using the Mini International Neuropsychiatric Interview (M.I.N.I.) [50]. Considering that anxiety or depressive disorders frequently co-occur in patients with AN [51], the presence of these comorbidities was not exclusionary. For HC, the exclusion criteria were having had a lifetime diagnosis of ED or obesity and/or a current DSM-5 diagnosed psychiatric disorder or obesity. For all participants, the exclusion criteria were being male, having an organic mental disorder, having had head trauma with a loss of consciousness for more than 2 min, having a learning or intellectual disability, being pregnant or currently breastfeeding, and any contraindication for magnetic resonance imaging (MRI) scanning.

The study procedures were carried out in accordance with the Declaration of Helsinki. The Clinical Research Ethics Committee of the Bellvitge University Hospital approved the study (PR319/20). Written informed consent was obtained from all participants before taking part in the study.

### Procedure

The assessment was performed in two separate sessions. Anthropometric and clinical variables, as well as blood samples to evaluate circulating eCBs concentrations (i.e., 2-AG and AEA), were collected during the first session. This was conducted before starting treatment in the AN group. Participants completed functional magnetic resonance imaging (fMRI) scanning during a second session. Patients with AN underwent assessments and fMRI scanning at the start of treatment.

#### Body mass index measure

The height of the participants was determined using a stadiometer. This information was entered into a Tanita Multi-Frequency Body Composition Analyzer BC-420MA (Tanita BC-420MA, Tanita Corp. Tokyo, Japan). This is a bioelectrical impedance analyzer and a non-invasive instrument that measures weight and estimates body composition. These data were used to calculate BMI.

#### Peripheral endocannabinoids measures

Blood samples were obtained after overnight fasting. Blood was centrifuged at 1700g in a refrigerated centrifuge (4 °C) over 20 min. Plasma was separated immediately and stored at −80 °C until eCBs (i.e., 2-AG and AEA) were analyzed, by liquid chromatography-mass spectrometry (LC/MS-MS) following a previously validated method [52].

### Neuroimaging analysis

#### Imaging data acquisition

Whole-brain resting-state fMRI (rsfMRI) data were obtained using a 3.0 Tesla clinical MRI scanner equipped with a 32-channel phased-array head coil (Intera Achieva Philips Medical Systems, Eindhoven, Netherlands). During an 8-minute sequence, participants were instructed to relax, stay awake, and lie still with their eyes open while observing a fixation cross. 240 whole-brain volumes were acquired using T2*-weighted echo-planar imaging (EPI) with a repetition time (RT) of 2000 msec, an echo time of 25 msec, and a pulse angle of 90°, in a 24-cm field of view (FOV) and an 80×80-pixel matrix, providing isotropic voxel sizes of 3 × 3 × 3 mm with no gap. A structural MRI scan was acquired for each participant. Specifically, a high-resolution T1-weighted anatomical scan was acquired to facilitate registration of the EPI data into standard MNI space, and for extracting individual global gray matter volume. A three-dimensional fast-spoiled gradient, an inversion-recovery sequence with 233 contiguous slices (repetition time, 10.43 msec; echo time, 4.8 msec; flip angle, 8°) in a 24-cm field of view, with a 320 × 320-pixel matrix and isotropic voxel sizes of 0.75 × 0.75 × 0.75 mm was used. In addition, participants had their heart rate frequency recorded with a BIOPAC MP150 data acquisition system and AcqKnowledge 4.4 software (BIOPAC Systems Inc., Goleta, CA).

#### fMRI preprocessing

fMRI data were processed and analyzed using MATLAB version 2019b (The MathWorks Inc., Natick, Massachusetts) and CONN toolbox version 2019b [53]. First, the BrainWavelet Toolbox was used to denoise all functional images. rsfMRI data underwent the following preprocessing steps: (1) functional realignment and unwarping, (2) slice-timing correction, (3) structural segmentation and normalization, (4) functional normalization, (5) ART-based identification of outlier scans for scrubbing according to previous recommendations [54], and (6) smoothing using a Gaussian filter (FWHM 8 mm). Physiological noise potentially disturbing the blood oxygen level-dependent (BOLD) signal (i.e., white matter, cerebrospinal fluid (CSF), and global BOLD time-series), as well as motion parameters (3 translational and 3 rotational axes) were introduced as confounders in an additional denoising step by the aCompCor [55] using the CONN toolbox [53]. Additional steps after denoising included the band-pass filtering of the BOLD time series (between 0.008 and 0.09 Hz), linearly detrending, and despiking to remove additional artifacts.

#### Seed-based functional connectivity analysis

We obtained a reward brain mask using Neurosynth inference maps (https://neurosynth.org). Neurosynth [56] is a meta-analytic neuroimaging database that uses a large series of previous studies to create empirical maps based on the probability that activation in specific brain regions would be associated with a specific term, such as “reward”. Choosing the “reward” term, a total of 922 studies were reported showing a forward-inference statistical map comprising several brain regions such as striatal regions, midbrain, cortices, hippocampus, amygdala, insulae, and cingulate, frontal, and intraparietal cortices (see Fig. S1a). The seed region within the reward system was selected by restricting the level of statistical significance of the empirical reward map. That is, we only included voxels with a high statistical probability (*p*_FDR_ = 0.00001) of association with the term “reward”. These resulted in a seed including 219 voxels within the ventral striatum/NAcc area (see Fig. S1a).

*First-level* (single-subject) maps were estimated in bivariate correlation analyses (Pearson’s *r*) of the resting-state BOLD time series, and NAcc-seeded connectivity maps were obtained for each subject. Heart rate frequency recorded during the rsfMRI was introduced as an individual regressor. A high-pass filter (128 s) was used to remove low-frequency drifts.

*Second-level analysis* (between-group effects) was carried out comparing NAcc functional connectivity between the AN group and the HC group. Given the significant age differences between groups, all analyses were controlled for this variable and corrected for multiple comparisons as recommended by Woo et al. [57] and Eklund et al. [58]. All derived differences in functional connectivity were analyzed under a statistical significance threshold satisfying a family-wise error (FWE) rate correction of p_FWE_ < 0.05 (spatial cluster extent).

### Statistical analyses of non-imaging data

Stata17 for Windows was used for the statistical analysis. The comparisons between groups were done with the analysis of variance (ANOVA) procedures, including age as confounding for the tests focused on the BMI, circulating eCBs concentrations, and eigenvalues from regions displaying significant between-group differences in functional connectivity. The assumptions of normality and homoscedasticity, required for the ANOVA, were met in this study (*p* > 0.05 in the Shapiro-Wilk tests and F-variance ratio test). The statistical power analysis yielded values ranging from β = 0.6 to 1 for mean comparisons.

The path analysis procedure explored the underlying associations between circulating eCBs, NAcc functional connectivity, and BMI. A multi-group model was tested including the diagnostic subtype as the group to assess the invariance of the structural coefficients between the AN and HC groups. Maximum likelihood estimation was used, and all parameters were freely estimated (any value was assumed and estimated by SEM). In order to obtain a more parsimonious model and increase statistical power, parameters with non-significant tests were deleted and then, the model was respecified and refitted. Goodness-of-fit was evaluated using standard statistical measures: chi-square test (χ^2^), the root mean square error of approximation (RMSEA), Bentler’s Comparative Fit Index (CFI), the Tucker-Lewis Index (TLI), and the standardized root mean square residual (SRMR). Adequate model fit was considered non-significant by χ^2^ tests if the following criteria were met: RMSEA < 0.08, TLI > 0.9, CFI > 0.9, and SRMR < 0.1 [59]. The global predictive capacity of the model was measured by the coefficient of determination (CD). In addition, due to the small sample size and the underpowered test for the SEM [60], in this analysis, relevant coefficients were considered for |standardized coefficient| ≥ 0.24.

## Results

### Sample description

Table 1 describes the study sample. The AN group was younger (*M*  = 22.89  ±  4.66) than the HC group (*M*  =  34.22  ±  7.78; *t* = *5.30*; *p* < 0.001). Likewise, the AN group had a lower BMI (*M*  =  16.28  ±  1.40) than the HC group (*M*  =  21.63  ±  2.06; *p* < 0.001). In the AN group, the mean age of onset was 17.11 years old, and the mean illness duration was 5.78 years. Likewise, an ANCOVA (adjusted for age) compared differences between groups in the 2-AG and AEA concentrations revealed no significant differences in 2-AG (*F*_(1,33)_ = 0.008; *p* = 0.930; |*d* | = 0.04) and AEA (*F*_(1,33)_ = 1.18; *p* = 0.285; |*d* | = 0.50) concentrations.

**Table 1:** Sample description.

	HC (*n* = *18*)		AN (*n* = *18*)		*p*	*|d* |
	*Mean*	*SD*	*Mean*	*SD*		
Age (years old)	34.22	7.78	22.89	4.66	<0.001*	**1.77**^**†**^
BMI (kg/m^2^)^a^	21.63	2.06	16.28	1.40	<0.001*	**3.04**^**†**^
Duration (years)	--	--	5.78	4.58	**--**	**--**
Onset (years old)	--	--	17.11	3.98	**--**	**--**
2-AG^a^	6.16	4.73	6.34	4.18	0.930	0.04
AEA^a^	0.24	0.09	0.20	0.08	0.285	**0.50**^**†**^

*HC* healthy controls, *AN* anorexia nervosa, *SD* standard deviation, *BMI* body mass index, *SD* standard deviation, *2-AG* 2-arachidonoylglycerol, *AEA* anandamide.

*Bold: significant comparison (*p* < 0.05). †Effect size in the moderate to high range.

^a^Comparison between groups: ANCOVA adjusted for age.

#### Between-group differences in NAcc functional connectivity

**Fig. 1:**
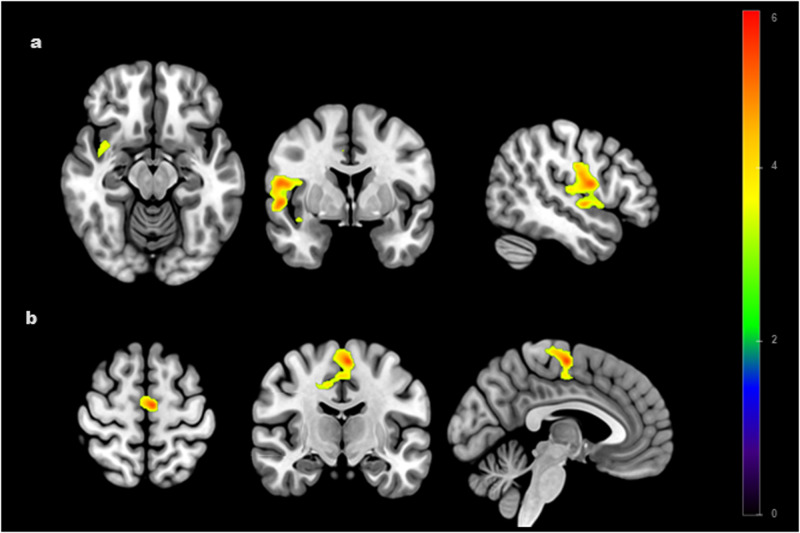
Between-group differences (AN < HC) in the functional connectivity of bilateral nucleus accumbens seeds. Figures (**a**, **b**) display lower functional connectivity between the nucleus accumbens and (**a**) the right insula (NAcc-insula; p_FWE_ < 0.001); (**b**) the left supplementary motor area (NAcc-SMA; p_FWE_ < 0.001) in the AN group compared to HC. Color bar represents t-values. Results are corrected and displayed at family-wise error (FWE) probability p_FWE_ < 0.05 threshold, cluster-extent. AN anorexia nervosa, HC healthy controls.

In comparison to HC, individuals with AN showed lower rsFC between the NAcc and the insula encompassing the adjacent temporal gyrus and operculum (p_FWE_ < 0.001) and the supplementary motor area (p_FWE_ < 0.001) (Fig. 1; Table 2).

**Table 2:** Second-level analysis showed reduced NAcc functional connectivity in the AN group compared to the HC group.

Functional connectivity	Region	MNI Coordinates (x, y, z)	ke^a^	t
AN < HC	Insula	54; −6; −2	1368	5.89
	Supplementary Motor Area	−4; −12; 62	605	4.54

*AN* anorexia group, *HC* healthy control.

A familywise error (FWE) probability (pFWE < 0.05) cluster-extent threshold was used.

Coordinates (x, y, z) are given in Montreal Neurological Institute (MNI) atlas space.

^a^Cluster extent in voxels.

#### Path analyses

**Fig. 2:**
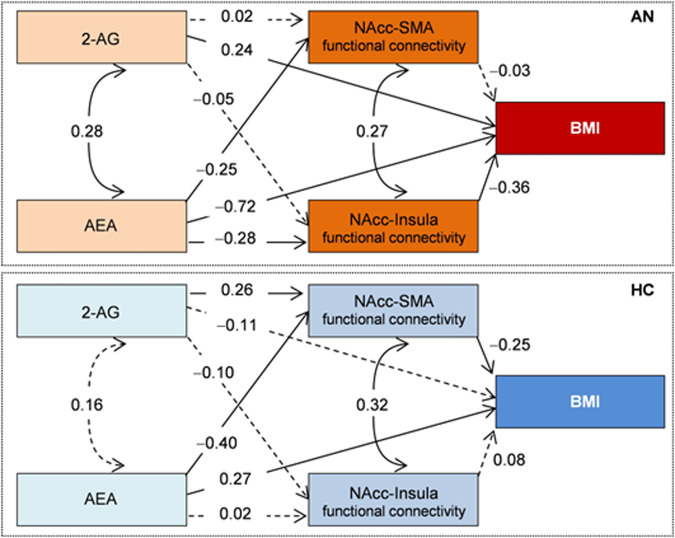
Path-diagram with the standardized coefficients (SEM model adjusted for age). AN anorexia nervosa, HC healthy controls, 2-AG 2-arachidonoylglycerol, AEA anandamide, NAcc nucleus accumbens, SMA supplementary motor area, BMI Body Mass Index. Continuous line: relevant coefficient. Dash line: non-relevant coefficient. Standardized coefficients ≥0.24 were considered relevant.

Our multigroup SEM obtained achieved adequate goodness-of-fit (χ2 = 2.15 [*p* = 0.341], RMSEA = 0.066, CFI = 0.989, TLI = 0.906, and SRMR = 0.038) and a global predictive capacity around 41% (CD = 0.411). A quasi-significant result was obtained in the joint test assessing the invariance of structural coefficients by group (χ2 = 16.28, *p* = 0.092), suggesting the existence of different structures within AN and HC groups. Figure 2 shows the path diagrams with the standardized coefficients. Within the AN group (marked in red in the figure), AEA and 2-AG showed a direct influence on BMI, whereas NAcc-insula functional connectivity had a dual role: a direct impact on the BMI and a mediational role on the relationship between AEA and BMI. Within the HC group (marked blue in the figure), AEA had a direct influence on the BMI, whereas NAcc-SMA functional connectivity had a dual role by contributing directly to BMI and mediating the relationship between AEA, 2-AG, and BMI.

## Discussion

The present study investigated NAcc functional connectivity and circulating eCBs concentrations in AN compared with HC, and their potential influence on BMI. In line with our hypothesis, we identified alterations in the functional connectivity patterns of the NAcc with the insula (NAcc-insula) and the supplementary motor area (NAcc-SMA) in individuals with AN compared to HC. Likewise, our results showed NAcc-insula and NAcc-SMA functional connectivity distinctively mediated the association between eCBs and BMI in each group.

Alterations in NAcc functioning have been widely reported in patients with AN [17, 27, 29–31, 33]. We identified NAcc-insula and NAcc-SMA hypoconnectivity in AN. The insula is the core of sensory, interoceptive, and gustatory processing regions [61], being part of the salience network (SN) [62], whereas the SMA plays an important role in motor planning and execution of voluntary movements, as well as in somatosensory processing, being part of the sensorimotor network (SMN) [62, 63]. In AN, aberrant communication between the insula and striatal regions has been associated with altered sensitivity to interoceptive and reward cues, which could be responsible for the development of maladaptive eating behavior and distorted perceptions surrounding hunger, satiety, and body weight and shape [32, 64, 65]. Insular dysfunction has been observed during the processing of taste and food rewards, which has been postulated as a possible mechanism underlying these behavioral and interoceptive disturbances in these patients [65]. Similarly, dysfunctional activity in the SMA has been related to alterations in body perception and severity of the disorder [66, 67]. For instance, a negative evaluation of body image in AN has been associated with lower functional connectivity of the SMA compared to HC [67]. Individuals in the acute stage of AN exhibited a lower SMA functional connectivity in comparison to both those who have recovered from AN and HC [66]. As speculative, this finding suggests that SMA functional connectivity could be affected in acute states of the disorder.

Concentrations of eCBs were not significantly different between groups, consistent with previous studies that observed no differences in 2-AG [13, 43] and AEA [44] concentrations. However, our findings revealed a specific interaction between eCBs and distinct NAcc functional connectivity in HC and AN concerning BMI. Higher AEA concentrations were associated with reduced NAcc-SMA functional connectivity and higher BMI in the HC group. The strength of NAcc-SMA functional connectivity in HC was a mediator in the association between both eCBs and BMI, but only AEA concentrations showed a positive direct association with BMI. A recent study also reported that circulating AEA concentrations influenced brain functional connectivity in HC evaluating changes during fasting and satiety [47]. However, in contrast to our findings, they observed that higher AEA concentrations were associated with increased functional connectivity in the caudate/NAcc with the insula and the anterior cingulate cortex [47]. Moreover, this study did not find a link between 2-AG and brain functional connectivity whereas we observed that higher 2-AG concentrations were associated with increased NAcc-SMA functional connectivity. Findings in HC support the notion that AEA and 2-AG may play distinct and independent roles in regulating BMI by exerting influence on reward and somatosensory circuits [24, 47]. While an increase in AEA concentrations would downregulate functional connectivity promoting weight gain, an increase in 2-AG would upregulate the strength of functional connectivity between NAcc-SMA counteracting weight gain. In other words, the influence of the NAcc-SMA functional connectivity on BMI could be distinctively regulated by AEA and 2-AG. These findings could have important clinical implications for the development of new therapeutic strategies aimed at maintaining a healthy weight, as a treatment for weight disorders.

In AN, both AEA and 2-AG were found to influence the association between the NAcc-insula and NAcc-SMA functional connectivity and BMI, a DSM-5 criterion of severity for AN [68]. Specifically, elevated AEA concentrations were directly associated with lower BMI, whereas 2-AG was associated with higher BMI. Based on the existence of compensatory mechanisms involving the eCB system [11, 48, 69], increasing AEA concentrations could represent an attempt to improve eating behavior and favor weight gain. However, the negative association between AEA and BMI in these patients could suggest resistance to this neuroendocrine signaling involved in the regulation of homeostatic and hedonic mechanisms related to food intake [70]. In contrast, the positive association between BMI and 2-AG may suggest that mutual regulation of AEA and 2-AG serves as a mitigator of severity in AN by modulating BMI. Consistent with this, studies using animal models have shown that infusion of cannabinoid agonists can elevate circulating 2-AG concentrations and CB1R activation in the NAcc, stimulating food intake and weight gain [38, 39].

Our SEM analysis in the AN group also showed a negative association between AEA concentrations and NAcc-insula and NAcc-SMA functional connectivity. In contrast to HC, NAcc-insula functional connectivity played a mediating role between AEA and BMI. That is, elevated AEA concentrations had a diminishing effect on NAcc-insula functional connectivity, which increased as a function of BMI. It is noteworthy that results derived from the association between AEA and NAcc-insula functional connectivity are opposite to those observed in the study by Martín-Pérez and collaborators [47], being argued that the influence of AEA on reward-related brain circuits would help to promote feeding in calorie-deprived situations [47, 71]. As this study was conducted in HC, our results might purpose the existence of a biological vulnerability pathway that could contribute to ignoring the somatosensory and interoceptive response to hedonic information in AN. Despite being speculative, the association of this described pathway with higher BMI in AN leads to the hypothesis of whether the existence of an underlying compensatory mechanism in the eCB system would involve an up-regulation of CB receptors in these regions. This up-regulation could be a response to the resistance of hedonic signaling mediated by AEA and the reduced NAcc-insula connectivity. Consistent with this rationale, in individuals with AN, an up-regulation of CB1R found in insular, frontal, and temporal regions suggested a compensatory response to altered circulating eCBs concentrations as a result of chronic starvation [48]. Given the preliminary nature of our results and the scarcity of evidence to dovetail with these findings, future studies should further confirm these assumptions.

This study should be interpreted considering some limitations. For example, its cross-sectional design does not allow the establishment of causal links. Likewise, there was a significant age difference between groups. Although this variable was controlled for in our analysis, future age-matched studies should be designed to minimize this potential age-related bias. In addition, due to the limited sample size used in this study, the mediation model exclusively used BMI as the clinical parameter of severity in order not to compromise the statistical power of the SEM analyses. Future studies with larger samples and further clinical parameters (in addition to BMI) could provide more evidence for these novel findings. Furthermore, the sample does not fully represent the population with AN because all patients were women recruited from a hospital setting. Finally, this study has not considered other variables of potential interest such as some hormonal factors (e.g., estrogen concentrations) [72, 73], or the well-known effect of physical activity on patients with AN that can modulate the eCB system tone [14]. However, the exclusive selection of the AN-R subtype was intended to eliminate the effect that purgative behaviors could potentially have on NAcc functional connectivity [33] and on circulating eCBs [43]. Future studies should examine the role of brain functioning and the interaction of eCBs in patients with EDs and purgative symptomatology. Additionally, this study also provides some noteworthy strengths. To the best of our knowledge, this is the first study exploring the association between deficits in brain reward function based on NAcc functional connectivity and circulating eCBs concentrations in patients with AN. From this perspective, the eCB system might be a potential target for treatment in AN and other EDs. Likewise, this study can also contribute to a deeper understanding of the neuroendocrine interplay between the eCB system and other neuroendocrine systems, considering the modulatory role that this system may have on the dopaminergic reward circuitry in AN.

## Conclusions

Dysfunctional connectivity between NAcc-insula and NAcc-SMA in AN may underlie alterations in the integration of interoceptive, somatosensory, and motor planning information, which could override responsiveness to hedonic information. Results from the multivariate SEM modeling indicate different association pathways between eCBs, functional connectivity, and BMI in AN and HC groups. These findings suggest that eCBs play a crucial role in influencing the relationship between brain networks and BMI in AN, shedding light on the neurobiological mechanisms underlying severity. The clinical implications of our results could contribute to the development of novel therapeutic strategies aimed at maintaining a healthy weight, as a treatment for weight and ED. Future research should investigate whether a potential causal relationship would exist between eCBs, NAcc connectivity, and the development of AN symptoms, such as restrictive eating behaviors.

### Supplementary information

Figure S1. NAcc bilateral seed from a reward brain mask obtained Neurosynth inference maps (https://neurosynth.org)
