# Supplementary material for: Exploring the influence of circulating endocannabinoids and nucleus accumbens functional connectivity on anorexia nervosa severity
Source: Mol Psychiatry. 2023 Sep 28;28(11):4793–800. doi: 10.1038/s41380-023-02253-2 (PMC10914605; doi:10.1038/s41380-023-02253-2)
Supplement: Supplementary file 1 — Figure S1. NAcc bilateral seed from a reward brain mask obtained Neurosynth inference maps (https://neurosynth.org) [file 41380_2023_2253_MOESM1_ESM.docx]

**SUPPLEMENTARY FIGURE**

***Figure S1.*** NAcc bilateral seed from a reward brain mask obtained Neurosynth inference maps (<https://neurosynth.org>)

**
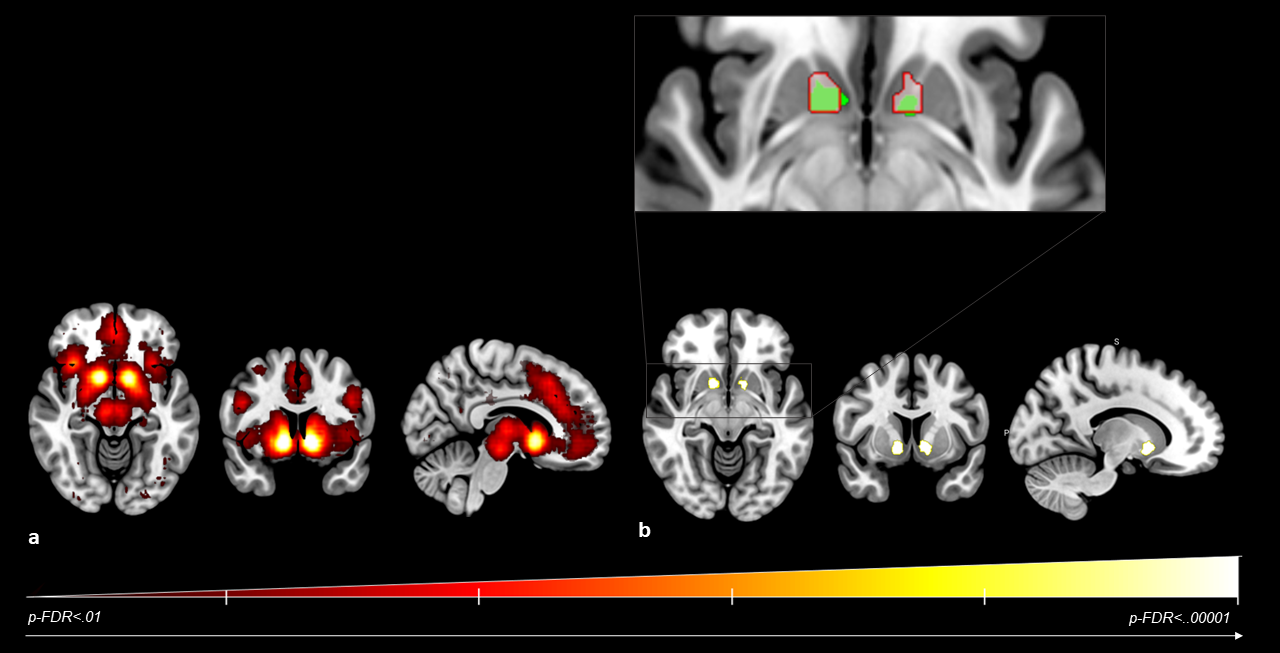
a.** Considering a total of 922 studies reporting the “reward” term, several brain regions such as striatal regions, midbrain, the hippocampus, amygdala, insula, cingulate, frontal, and intraparietal cortices were included in the empirical mask obtained from Neurosynth inference maps (family discovery rate (FDR) correction of p_FDR_<.01). To obtain the nucleus accumbens (NAcc) mask, statistical significance was restricted to p_FDR_<.00001. **b.** The final NAcc mask, comprised of 219 voxels, overlapped with the automated anatomical labeling (AAL3) atlas to confirm the location of the obtained seed. The green seed represents the NAcc anatomical template from the AAL and the white mask with red boundaries represents the NAcc Neurosynth mask.
